# 

**Published:** 1888-01

**Authors:** 


### BOOK NOTICES.

THE TWO WORLDS.—A journal devoted to spiritualism, occult science, ethics, religion and freedom, weekly, 61 George St., Chatham Hill, Manchester, England. Subscription price $2 per annum. Edited by Mrs. Emma Hardinge Brittan, than whom, no platform lecturer or writer is better and more favorably known in the United States. For many years Mrs. Brittan has been a constant and fearless advocate as well as exemplar, of that moral and intellectual freedom which grows with the knowledge of the truth, as opposed to the slavish dogmas of worn out systems and creeds, founded in superstition and nursed by the bigots that still linger about the dying embers of a false theology, that belittles the universal God, in proportion as it exalts the powers of an imaginary satan, who counteracts the divine purpose with ruinous cunning and insolent bravado.

May your good work go forward as it deserves. With this sentiment we give you our hand across the waters, "Two Worlds."

THE SOUL.—Volume 1, Number 1, monthly. Facts Publishing Co., Boston, Mass., $1.50 per annum. The above is a new 30 quarto pp. monthly edited by L. L. Whitlock, formerly editor of "Facts," of which the present monthly is an outgrowth. As indicated by its title, this publication is destined to enter a broader field and take a higher intellectual range, than one devoted to the recitation of mere facts, however interesting and marvelous, would be likely to attain.

WIDE AWAKE.—The holiday number of this journal is an excellent specimen in matter, typography and illustrations, particularly adapted to the young. Published by D. Lathrop Co., Boston, at $2.40 a year, or 20 cents a number.

We welcome to our table the following new exchanges, all excellent of their kind :
MENTAL SCIENCE MAGAZINE, monthly. 141 La Salle St., Chicago, Ill. $1 a year.
ANNALS OF GYNECOLOGY, monthly. Rockwell and Churchill, Boston. $1.

THE CENTURY.—This excellent publication still holds the lead of American magazines. There is no flagging in its Lincoln and War articles, which occupy the front line of its onward march. CENTURY CO., 14th St., N. Y.

